# UV-Enhanced Sacrificial Layer Stabilised Graphene Oxide Hollow Fibre Membranes for Nanofiltration

**DOI:** 10.1038/srep15799

**Published:** 2015-11-03

**Authors:** J. Y. Chong, N. F. D. Aba, B. Wang, C. Mattevi, K. Li

**Affiliations:** 1 Department of Chemical Engineering, Imperial College London, London SW7 2AZ, UK; 2 Department of Materials, Imperial College London, London SW7 2AZ, UK

## Abstract

Graphene oxide (GO) membranes have demonstrated great potential in gas separation and liquid filtration. For upscale applications, GO membranes in a hollow fibre geometry are of particular interest due to the high-efficiency and easy-assembly features at module level. However, GO membranes were found unstable in dry state on ceramic hollow fibre substrates, mainly due to the drying-related shrinkage, which has limited the applications and post-treatments of GO membranes. We demonstrate here that GO hollow fibre membranes can be stabilised by using a porous poly(methyl methacrylate) (PMMA) sacrificial layer, which creates a space between the hollow fibre substrate and the GO membrane thus allowing stress-free shrinkage. Defect-free GO hollow fibre membrane was successfully determined and the membrane was stable in a long term (1200 hours) gas-tight stability test. Post-treatment of the GO membranes with UV light was also successfully accomplished in air, which induced the creation of controlled microstructural defects in the membrane and increased the roughness factor of the membrane surface. The permeability of the UV-treated GO membranes was greatly enhanced from 0.07 to 2.8 L m^−2^ h^−1^ bar^−1^ for water, and 0.14 to 7.5 L m^−2^ h^−1^ bar^−1^ for acetone, with an unchanged low molecular weight cut off (~250 Da).

Graphene oxide (GO), a functional derivative of graphene, has been considered as a promising membrane material and GO membranes have shown to be effective separation barriers in environmental and chemical engineering application^1^,^2^,^3^,^4^,^5^,^6^,^7^,^8^. GO flakes have an effective thickness of about 5 to 10 Å^9^,^10^ and a plane width of few to tens microns. With the large number of oxygenated surface functional groups and the high area-to-thickness ratio, GO flakes can be assembled to form multilayer thin films or membranes by stacking on top of each other in parallel^11^,^12^. Although GO membranes thicker than 1 μm were found to be impermeable to gases including helium^4^, GO membranes as thin as a few tens of nanometres were shown to have high gas permselectivity at considerable fluxes^3^,^5^. GO membranes have also shown potential in aqueous solution nanofiltration to retain molecules with molecular weights of several hundred dalton^13^,^14^,^15^, and have been tested for pervaporation to separate water from organic solvents^16^,^17^. Water molecules were found to be able to travel fast within the interlayer space between GO flakes, and the separation of molecules in GO membranes was proposed to be due to the interlayer distance between the GO laminates^4^,^18^.

Although GO membranes have shown great potential in separation processes, it is still far from real industrial applications due to unsolved engineering problems. In practice, ultrathin GO membranes need to be supported on a substrate for large scale applications to withstand high pressure difference across the membrane. So far, planar polymeric or ceramic supports have been used for GO membranes in most laboratory studies^3^,^5^,^13^, but they are difficult for mass production and their membrane area to volume ratio is limited. Towards engineering perspectives, much effort has been put on preparing GO membranes on hollow fibre supports, often with a diameter of 1 mm or smaller, in order to achieve the highest membrane area to module volume ratio (up to 9000 m^2^/m^3^).

Of all the types of hollow fibre substrates, ceramic hollow fibre is an excellent candidate due to its robust mechanical properties and inert chemical nature, which enable it to work under extreme conditions. Furthermore, recent research has revealed that ceramic substrates release multi-valent cations, which can help to crosslink GO flakes and make GO membranes stable in water; GO membranes prepared with polymeric substrates, in contrast, are unstable in water^19^. The use of GO/ceramic hollow fibre composite membrane was recently reported for dehydration of dimethyl carbonate and for nanofiltration^17^,^20^. However, our recent research revealed that supported GO membranes are unstable in dry state, at least on ceramic hollow fibre substrates^20^. This is due to the shrinkage generated during the drying course, which produces enormous tensile stress in the GO membrane and leads to defects. To tackle the instability problem, GO hollow fibre membranes can be reserved in water after initial drying to avoid further shrinkage, so that fatal defects can be prevented^20^. Such a strategy was shown to work well, but the resultant membranes could only be used for limited wet separation processes such as nanofiltration and pervaporation. Besides, tailoring the GO membranes via post-treatments for different purposes would be difficult since many post-treatments are only possible in a dry environment. Thus, effective approaches to stabilise GO hollow fibre membranes in dry state are needed to expand the applications of GO membranes.

Here we present an effective approach to solve the stability problem by using a sacrificial layer during the membrane fabrication. The sacrificial layer is used to create an extra space between the ceramic hollow fibre substrate and the GO membrane. When the sacrificial layer is washed off, the resultant gap in between allows the GO membrane to shrink freely without introducing stress. The idea is schematically shown in Fig. 1. With the improved stability of the GO membrane, potential post-treatments can then be used to alter the properties of the membranes. For example, we show in this study that UV treatments on the GO hollow fibre membranes could induce the formation of controlled microstructural defects in the membrane and dramatically reduce the water contact angle of the membrane surface by increasing the surface roughness factor. The UV post-treatment has greatly enhanced the permeability of the GO membranes, enabling their successful usage for nanofiltration purposes.

## Results and Discussion

### Sacrificial layer and Stability

**Figure 2:**
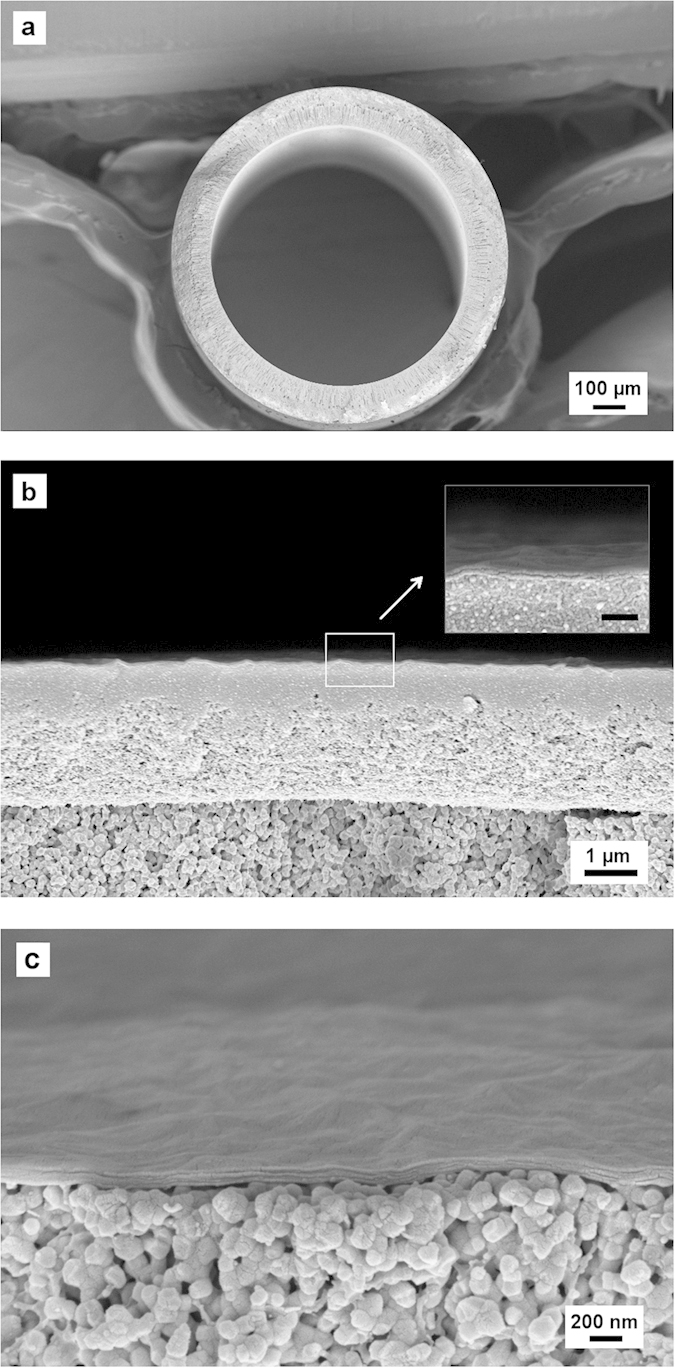
(**a**) The whole view of the cross section of a YSZ hollow fibre. (**b**) An SEM image showing the GO/PMMA/YSZ tri-layer structure, where the sacrificial PMMA layer is sandwiched in the middle by the top GO layer and the bottom YSZ substrate. (Scale bar = 200 nm in the smaller image) (**c**) An SEM image showing the GO/YSZ structure after the PMMA layer was washed by acetone.

The idea of sacrificial layer aided GO membrane fabrication was implemented with porous yttrium stabilised zirconia (YSZ) hollow fibre substrates (Fig. 2(a)). The YSZ hollow fibre support has an outer diameter of 1 mm, and a high pure water flux of 1200 L m^−2^ h^−1^ bar^−1^, which ensures a negligible permeation resistance in the substrate compared to the GO membrane layer. The substrate also has a smooth surface, in which the average surface pore size is about 80 nm, and the surface roughness is about 200 nm. PMMA, a cheap and widely used polymer was used as the material for the sacrificial layer. A PMMA layer was coated on the ceramic hollow fibre substrate with dip coating and underwent a phase inversion process to form a porous PMMA structure. The porous PMMA layer acted like a permeable membrane enabling GO deposition using vacuum filtration. During the vacuum-facilitated flow directed deposition process, GO flakes self-assembled, in parallel to each other, on top of the PMMA sacrificial layer forming a GO membrane layer. After the GO deposition, the PMMA sacrificial layer was washed off by immersing the hollow fibre in acetone. This could be done easily without affecting the quality of the GO layer due to the inert property of GO membranes in organic solvent. A gap was then created between the substrate and the GO membrane, allowing the GO membrane to shrink freely without introducing stress when drying.

Figure 2(b) shows a scanning electron microscope (SEM) image of a GO/PMMA/YSZ hollow fibre, it can be seen clearly that the PMMA sacrificial layer, about 3 μm thick, is sandwiched between the thin GO membrane and the YSZ substrate. As GO membranes would experience shrinkage of about 1% when drying^20^, the optimum thickness of a PMMA layer for perfectly stress-free shrinkage is 5 μm for the hollow fibres (OD = 1 mm) used in this study. However, GO membranes can withstand a high tensile stress, up to a maximum fracture stress of 120 MPa, and has a maximum strain of 0.6% at the fracturing point^12^. Therefore, the minimum thickness of the PMMA layer required to avoid fracture is approximately 2 μm. In our study, PMMA thickness greater than 3 μm was sufficient to stabilise GO hollow fibre membranes, and wrinkles due to excessive space were observed when the sacrificial layer was thicker than 5 μm. Figure 2(c) shows that the PMMA layer can be completely removed after leaching with acetone, and the GO membrane sits tightly on the YSZ substrate with a well-packed layered structure.

**Figure 3:**
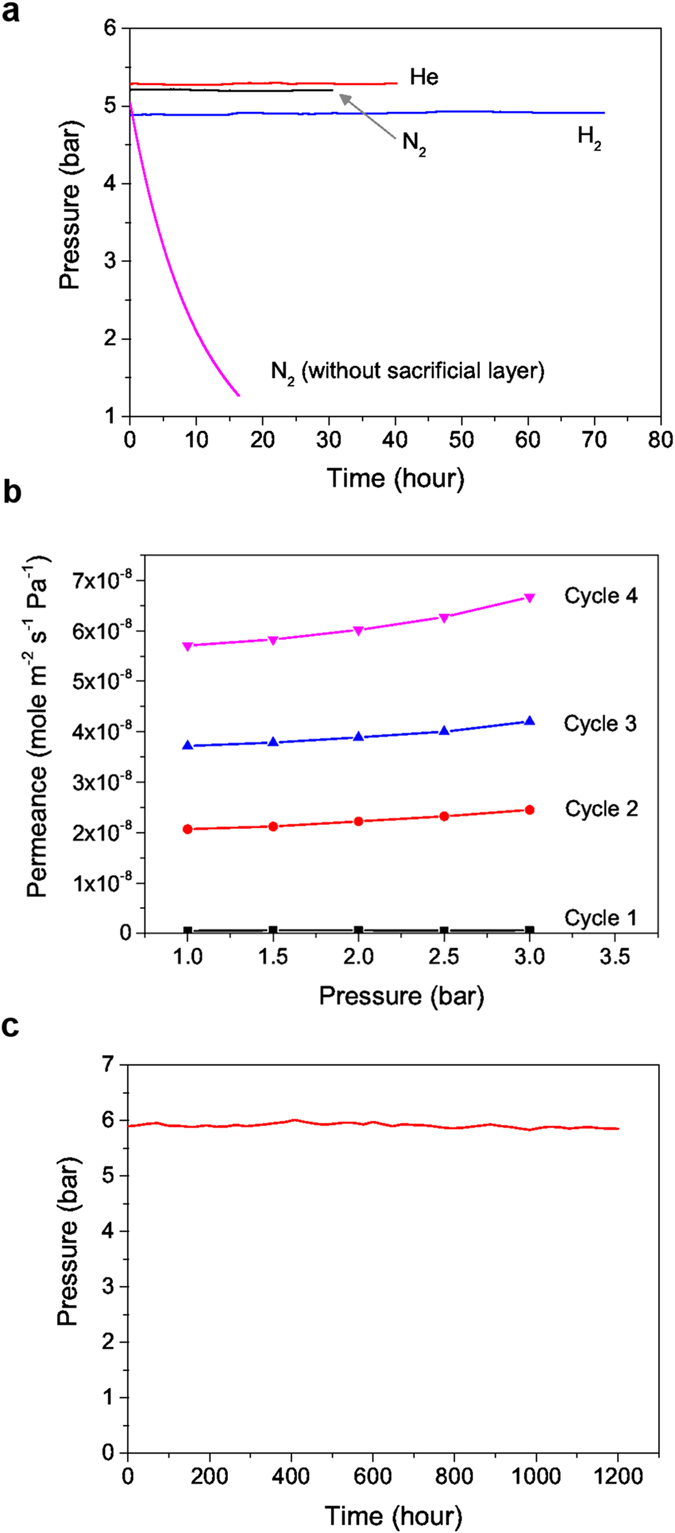
(**a**) The results of gas tightness tests of the GO/YSZ hollow fibre membrane prepared with a sacrificial layer. The GO membrane was about 700 nm thick. The tests were conducted with the sequence of N_2_, He and then H_2_. The test result of a GO/YSZ membrane without using a sacrificial layer is also presented for comparison. (**b**) The result of repeated N_2_ gas permeation tests of a dry GO/YSZ hollow fibre membrane without using a sacrificial layer. The timeline of each test cycle was 1 day, 2 days, 4 days and 7 days after the GO membrane was dried for cycle 1, cycle 2, cycle 3 and cycle 4, respectively. (**c**) The result of a long term stability test with O_2_ on a 150 nm-thick sacrificial layer stabilised GO/YSZ hollow fibre.

The gas-tightness property of the GO hollow fibre membrane was tested after the removal of the PMMA layer. For GO membrane with a thickness of 700 nm, the membrane was impermeable to various gases including N_2_, He and H_2_, as shown in Fig. 3(a). The test was run for 160 hours in total and no leakage was observed, demonstrating that the GO hollow fibre membrane was very stable during the test for all different gases tested. The gas impermeable property of GO membranes was similar to the results shown by Geim *et al.* where a free standing GO paper was tested^4^. Unlike their studies, we tested the GO membranes at a higher pressure, around 5 bar, which is closer to real gas separation operating conditions. We found that the membranes were still stable under such a high pressure. Similar GO hollow fibre membranes without a PMMA sacrificial layer allowed gases to leak quickly with a permeance higher than 8.0 × 10^−9^ mol m^−2^ s^−1^ Pa^−1^ (the pressure drop result is also presented in Fig. 3(a) for comparison). Repeated tests with the same membrane but at different times showed the gas permeance increased over time, implying continuous development of defects in the membranes (Fig. 3(b)). The unstable nature of the GO hollow fibre membranes has been discussed in detail in our previous work^20^. We further tested the long-term stability of the PMMA sacrificial layer stabilised GO membranes (Fig. 3(c)). A GO hollow fibre membrane with a thickness of 150 nm was tested with O_2_ for 1200 hours at a pressure of around 6 bar and the pressure did not decline throughout the entire test. The long-term test confirms that the stabilised GO hollow fibre membrane was extremely stable, and did not degrade within the 50-day test under a high pressure. It also verifies that a defect-free GO membrane is impermeable to gases, given that our test was under the most stringent conditions so far.

The gas-tightness tests showed GO hollow fibre membranes can be stabilised in the air after overcoming the shrinkage problem with a sacrificial layer. However, the resultant defect-free microstructure leads to undesired performance changes in nanofiltration. The interlayer distance of GO membranes vary from 0.7–1.4 nm depending on the water content^4^,^18^, and the larger interlayer distance in water enables them to be useful for nanofiltration. In our previous study in which GO membranes were stabilised in water, water and acetone permeated through the membranes, with a thickness of 1.5 μm, at a considerable flux of 1.04 and 6.35 L m^−2^ h^−1^ bar^−1^, respectively, whilst completely rejecting dye molecules larger than 327.3 Da^20^. However, for the GO membranes stabilised by a sacrificial layer, the water permeation was extremely low, at only 0.074 L m^−2^ h^−1^ bar^−1^ through a 150 nm thick GO membrane. The permeability was two orders of magnitude lower than the membranes in our previous study^20^. The acetone permeation of the stabilised GO membrane was also very low, only 0.14 L m^−2^ h^−1^ bar^−1^. The dramatic reduction of the permeability is believed to be the consequence of the elimination of microstructural defects in the GO membranes, which can act as shortcuts for the transport of liquids and thus greatly reduces the permeation resistance in the non-stabilised membranes. However, the permeability of the stabilised GO membranes can be recovered or even further improved by post-treatments. In this research, we used UV radiation as an example to illustrate the possibility of tuning the property of the stabilised GO membranes.

### UV Post-treatment and Nanofiltration

**Figure 4:**
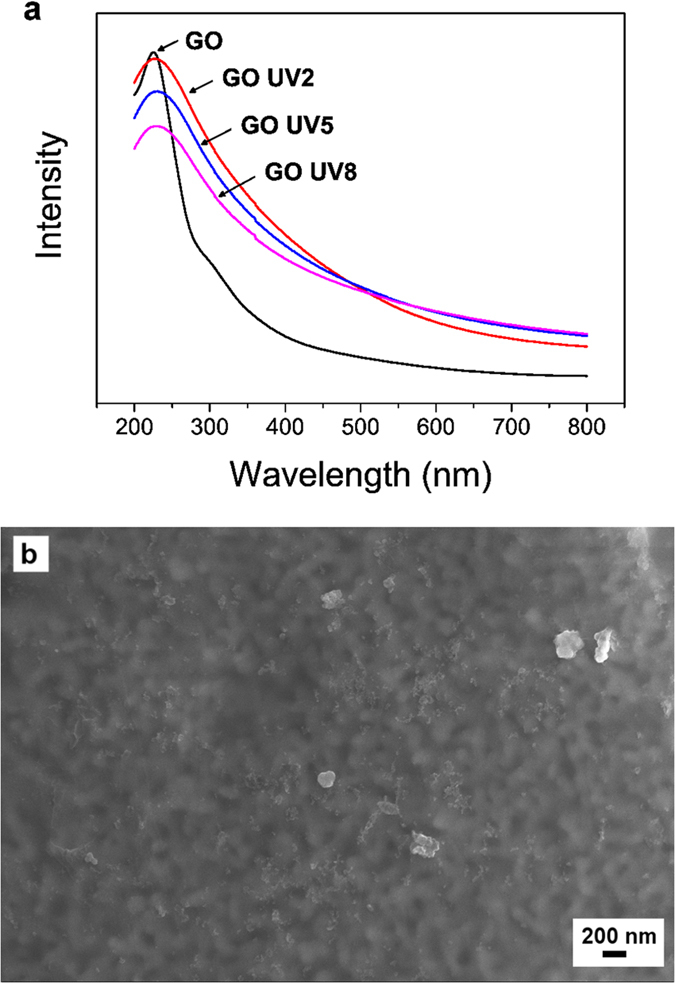
(**a**) UV spectrums of thin GO layer coated on a quartz glass before and after UV treatments. (**b**) SEM image of GO membrane with 2 hours of UV treatment without metal plasma coating.

By using a sacrificial layer, stable GO hollow fibre membranes were successfully determined and these membranes can be post-treated in dry state to improve their performance. UV radiation has been used to modify the property of GO flakes, in which it can mildly reduce the functional groups on GO^21^,^22^. In our study, we post-treated the stabilised GO hollow fibre membranes with UV radiation and we found that it also led to similar changes to dry GO membranes in the air. Visually, the GO membranes turned from light brown to dark grey after UV treatment; this is close to the colour change of GO membranes reduced at elevated temperature under inert atmospheres. To further confirm the reduction of GO membranes after the exposure of UV light, GO suspension was drop-casted on a quartz glass to form a thin GO layer. The GO coated quartz glass was exposed to UV light for 2, 5 and 8 hours, and the change in functional groups of the GO was monitored using a UV-spectrometer. The UV spectrums are shown in Fig. 4(a). Before the exposure to UV light, the UV spectrum of the GO coating showed a peak at wavelength ≈225 nm, which could be assigned to the π → π* transition of aromatic C–C bonds, and a shoulder at ≈300 nm, which corresponded to n → π* transition of C=O bonds^23^,^24^. After the exposure of UV light, the shoulder was no longer observed and it could be due to the reduction of GO. The change in the UV spectrum coincided with the UV spectrum of reduced GO (rGO)^25^. However, there was no obvious shift of the peak at ≈225 nm, compared to highly reduced rGO in which a peak shift to ≈270 nm was commonly observed, as the reduction of GO under the UV light could be relatively mild in our case^25^. Besides, the reduction of GO could also be observed when SEM images were taken. For GO membrane without UV treatment, metal plasma coating was required to observe the membrane surface. However, the electrical conductivity of GO membranes became higher after UV treatment and SEM images could be taken without metal plasma coating. As shown in Fig. 4(b), the GO layer looked transparent under SEM and the hollow fibre substrate underneath could be observed. The higher conductivity can be attributed to the more conductive rGO area in the membranes after the mild reduction.

**Figure 5:**
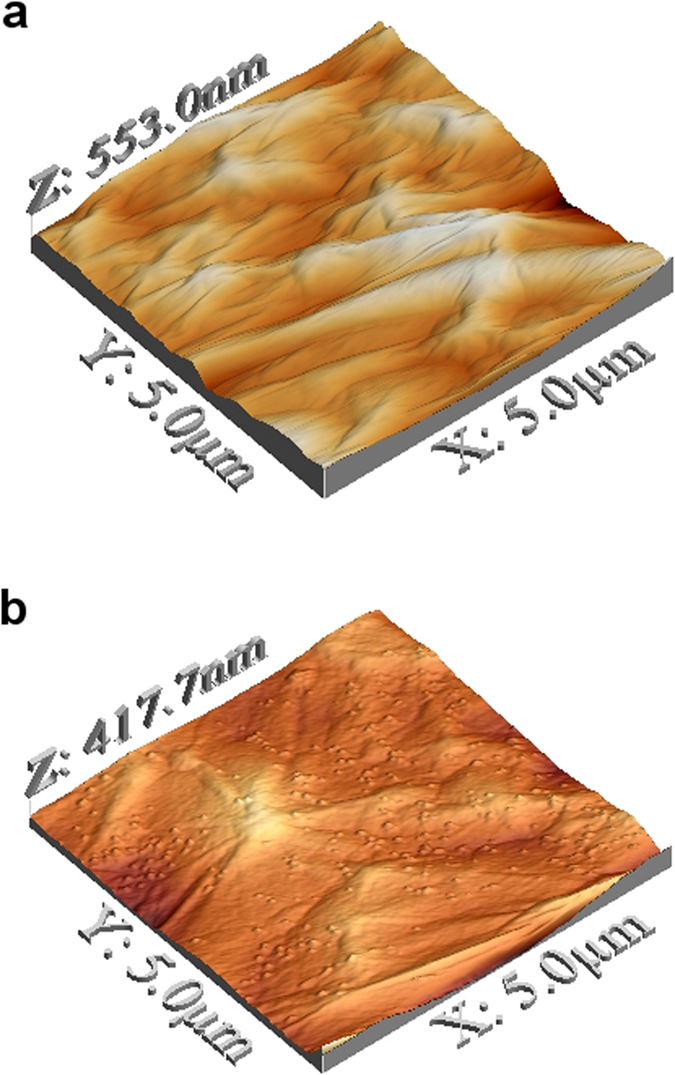
AFM images of GO membranes on a PES filter (**a**) before UV treatment; (**b**) after 2 hours of UV treatment.

**Figure 6:**
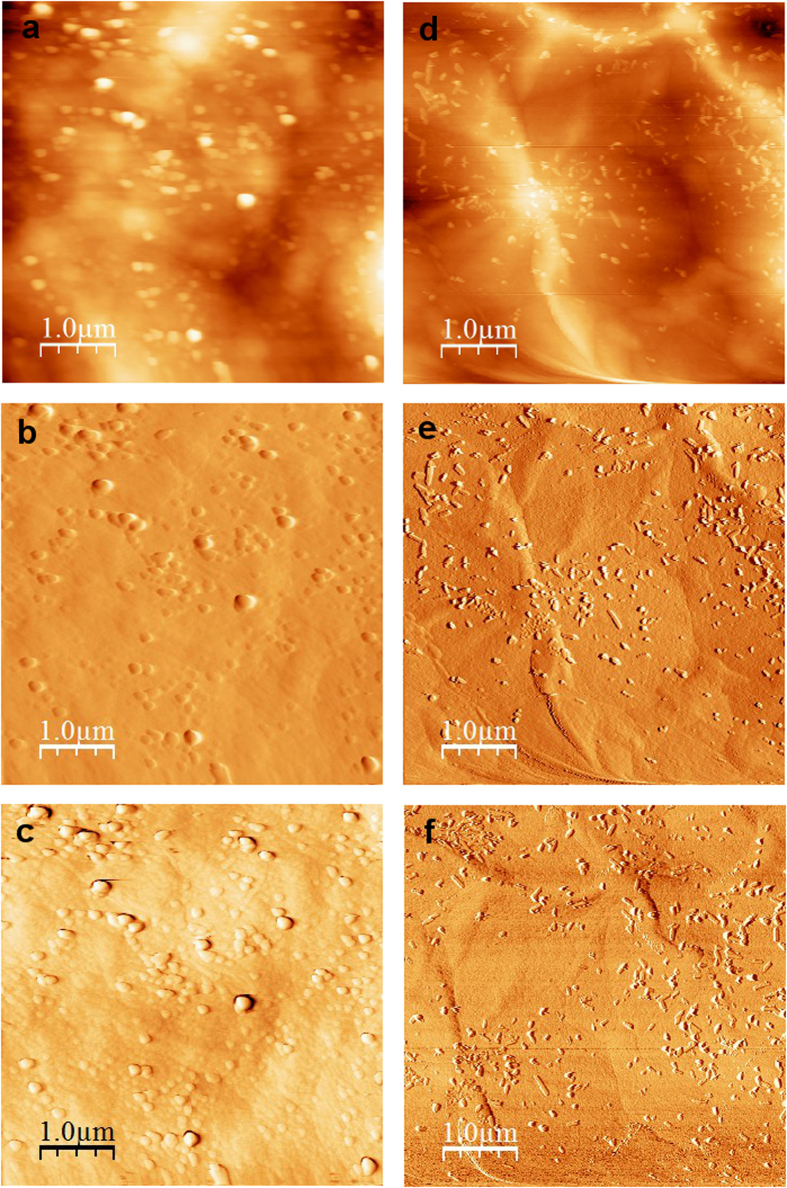
AFM images of the surface of GO membrane on PES substrate after (**a-c**) 5 and (**d-f**) 8 hours of UV treatment. (**a**,**d**) are the topology images, (**b**,**e**) are the gradient images and (**c**,**f**) are the phase images of the membrane surfaces.

The change of surface morphology was examined with AFM, and the images of the surface are presented in Fig. 5(a,b). It can be observed that after 2 hours of UV treatment, many small features appear intensively on the surface of the GO membrane. These features resemble small bumps on the membrane surface, and they were also observed on other UV-treated membranes with different exposure time (Fig. 6). Phase analyses of the AFM images (Fig. 6(c,f)) reveal that these small bumps are of the same material as the rest of the surface, thus excluding the possibility that they are due to contamination from the environment during UV treatment, but originated from GO itself. These bumps on the surface could be created by local stress near the surface resulting from the mild reduction of GO. We measured the water contact angle of the GO membranes before and after UV treatment (Supplementary Information Fig. S1). Before UV treatment, the GO membrane showed a contact angle of 39.5°, which is concurrent with the finding in other studies^14^,^26^. The contact angle reduced to zero after UV treatments (2 hours or longer). The effects of surface morphology on contact angle are commonly described using the Cassie-Baxter model (Equation 1), which assumes the contacting area is a mixture of liquid-solid interface and liquid-air interface^27^:


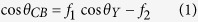


where 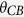 is the apparent contact angle, 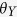 is the contact angle on dense smooth surface (the Young’s contact angle), *f*_*1*_ is the area of the liquid-solid interface, and *f*_*2*_ is the projected area of the liquid-air interface. From this equation, when the Young’s contact angle is smaller than 90 °, the apparent contact angle will reduce when the roughness factor, *f*_*1*_*/f*_*2*_ increases. The roughness factor is determined here by the intensity of the small features on the membrane surface.

**Figure 7:**
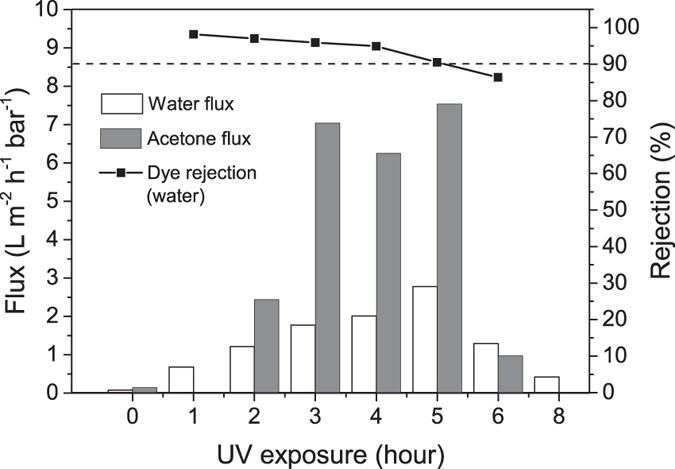
Nanofiltration performance of GO membranes with different UV exposure times. The white columns present the pure water permeation while the grey columns show the permeation of acetone. The rejections of methyl red (M_w_ = 269.3 Da) in aqueous solution are shown with square dots.

Nanofiltration performance of the UV-treated membranes was also tested. After exposure to UV for certain period of time, the permeability of the stabilised GO hollow fibre membranes increased substantially, as shown in Fig. 7. The water and acetone permeation fluxes increased along with the exposure duration, and reached the maximum values of 2.8 and 7.54 L m^−2^ h^−1^ bar^−1^, respectively, after 5 hours of UV treatment, whilst dye rejection tests showed these membranes remained highly selective with a rejection rate of >90% to methyl red (M_w_ = 269.3 Da). However, when the GO membranes were exposed excessively to UV light, an adverse effect was observed. Instead of further increasing, the fluxes of water and acetone decreased when the UV exposure time was longer than 5 hours. The permeation flux of water and acetone decreased to 1.3 and 1.0 L m^−2^ h^−1^ bar^−1^, respectively, when the GO hollow fibre was exposed to UV light for 6 hours. The water flux further decreased to 0.42 L m^−2^ h^−1^ bar^−1^ when the membrane was exposed to UV light for 8 hours. Besides, the rejection of GO membranes to methyl red was also lower, only 86.4% when the UV exposure time was 6 hours, compared to above 90% in cases where membranes with shorter hours of UV treatment. Rejection tests to multivalent cation Mg^2+^ were also conducted with the UV treated membranes (5 hours). The rejection rate was 78.4%, which indicates that the membranes can effectively remove multivalent heavy metal ions in waste water.

**Figure 8:**
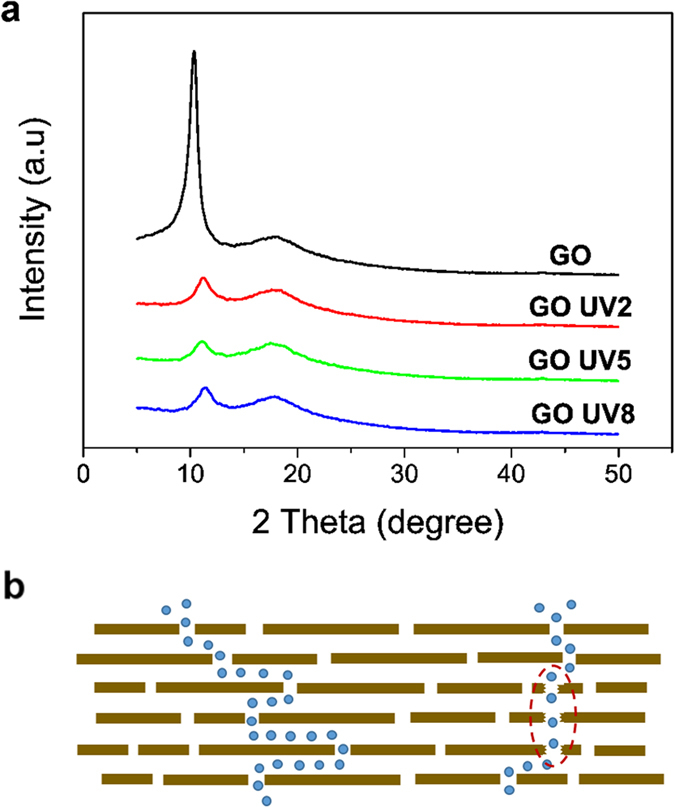
(**a**) XRD patterns of flat-sheet GO membranes made on a PES microfiltration filter before and after UV treatments. The interspace between GO flakes in the membranes did not change at 0.8 nm. The broad peak around 18° is from the substrate, not from the GO membrane. (**b**) Microstructural defects in GO membrane formed as a result of reduction during UV treatment, serve as shortcuts for the liquid permeation process.

It is of great interest to investigate how the mild reduction of the GO membranes led by UV treatment could cause a dramatic improvement in the permeability. XRD results (Fig. 8(a)) showed the interlayer distance of about 0.8 nm between the GO planes in the membranes was not affected by the UV treatment and only a marginal peak shift was observed. However, the intensity of the peak corresponding to the interlayer distance was considerably weaker after UV treatment, which could be due to the imperfection of GO laminar structure or defects generated in the GO membrane during UV treatment. The formation of microstructural defects by UV can be evidenced by the degradation of the dye rejection rate when the UV exposure duration exceeded 5 hours. The change of microstructure of the membrane after UV treatment may have contributed to the enhanced permeability^20^,^28^. When liquid transports in the bulk of the membrane, the transport resistance is mainly affected by the length and the size/diameter of the pores. We recall that the permeability of GO membranes can be greatly affected by the presence of microstructural defects generated during the drying process for the membranes without using a sacrificial laye^20^. The microstructural defects serve as shortcuts for the permeation process (Fig. 8(b)), reducing the length of the transport passage and therefore improved the membrane permeability. For the membranes without using a sacrificial layer, defects developed from drying are difficult to control, especially for very thin GO membranes. Fatal defects crossing over the membrane can be easily induced in a short time during drying, which will cause the membrane to lose its selectivity. But in this study, the process of producing microstructural defects by UV is a much slower one, and it stops once UV exposure is stopped, thus it is more controllable to avoid fatal defects.

Although UV exposure can lead to the formation of microstructural defects which improve the water permeation of GO membranes, the reduction of oxygen-containing surface functional groups may produce an opposite effect after prolonged UV treatment, causing a decrease in water flux after 6 and 8 hours of UV exposure. The adverse effect can be interpreted as the result of locally reduced hydrophilicity at pore entrances due to reduction, which hampers the entry of water into the pores, even when the wettability on the surface remains high. A similar situation was observed when GO hollow fibre membrane was annealed at elevated temperature in vacuum. Microstructural defects were generated in the membrane after thermal annealing due to further shrinkage of GO membranes^20^ and the loss of oxygen containing surface functional groups from GO^29^. However, the water permeation of the annealed GO membrane was still very low, 0.13 L m^−2^ h^−1^ bar^−1^, as the loss of surface functional groups, especially near the pore entrances, may have had a larger effect in preventing water from entering the membrane.

## Conclusions

GO membranes supported by ceramic hollow fibres are unstable primarily due to shrinkage during drying. This stability issue can be well addressed by using a sacrificial layer, which creates a gap between the GO membrane and the substrate, allowing the GO membrane to shrink freely in the absence of mechanical stress. The GO membrane fabricated with a sacrificial layer was stable in a long-term stability test over 1200 hours in dry state. Possibility of post-treatments of the GO membranes in dry state is exampled by UV treatment, which can induce the formation of microstructural defects and greatly enhance the permeability without losing the molecular sieving property. Such membranes show high permeation fluxes of water and acetone compared to commercial nanofiltration membranes, and reject multivalent cations and molecules larger than 250 Da, implying a promising practical potential usage for wastewater treatment and organic solvent nanofiltration. Additionally, given the relatively large thickness of the membranes in this study, there is still space to further improve the performance of the hollow fibre GO membranes. With the membrane permeability determined in this study, the flux of GO membranes is projected to increase significantly if the membrane thickness could be substantially reduced to, say, below 20 nm, which is achievable for GO membranes as demonstrated in other studies^3^,^5^.

## Methods

### Preparation of GO/YSZ hollow fibre membranes and GO flat sheet membranes

GO dispersion was prepared through a modified Hummer’s method^30^,^31^. The GO dispersion was centrifuged to control the size of GO flakes, which was determined to be 5–10 microns from the SEM. Yttrium stabilised zirconia (YSZ) hollow fibres were fabricated through a combined phase-inversion/sintering process, and the green fibres were sintered at 1150 °C to gain strength^32^. To deposit GO membrane onto the surface of YSZ hollow fibres, the hollow fibre was immersed in a GO dispersion with one end sealed and the other end connected to a vacuum pump. When vacuum was applied from the lumen of the fibre, water was sucked into the fibre and the water flow directed GO flakes to the outer surface of the hollow fibre substrate. GO membrane was formed by stacking the flakes layer by layer, with the aid of pressure difference and water permeation. The thickness of the GO membrane was controlled by changing the initial concentration of the GO dispersion and the duration of filtration.

For GO hollow fibre membranes with a poly(methyl methacrylate) (PMMA) sacrificial layer, the YSZ hollow fibre substrate was first coated with a PMMA (15 wt%)/NMP (85 wt%) solution by dip-coating at a controlled dipping and withdrawing rate. The coated polymer solution layer was then immersed into water to form a porous polymer layer via a phase-inversion process. A GO membrane layer was then deposited onto the PMMA layer through the same method as described above. After initial drying in ambient air for 15 minutes, the GO/PMMA/YSZ fibre was immersed in ethanol to extract water and form connections between the GO flakes, and then into acetone for at least 2 hours to leach the PMMA layer. After drying in ambient air, the GO hollow fibre membrane was assembled on a stainless steel holder for tests.

Some flat sheet GO membranes were also prepared for a more accurate characterization of the membranes. The flat sheet membranes were synthesized using the same vacuum-facilitated filtration method on a Supor® polyethersulfone (PES) microfiltration membrane, with a pore size of 0.2 μm.

### UV light treatment

Sacrificial layer stabilised GO hollow fibre membranes were exposed to UV light generated from a Pen-Ray® Mercury lamp in a black box. The UV light source has a wavelength of 230 nm at an intensity of 5.4 W/cm^2^. GO hollow fibre membranes were divided to four surface sides and each side was exposed to the UV light at a certain exposure time.

### Gas-tightness/gas-permeation tests and dye-rejection tests

The gas-tightness/gas permeation tests were conducted with a setup made of Swagelok VCR® fittings. The setup is equipped with a high-precision pressure transducer (Mensor CPT6180) with reading uncertainty of 0.01%. A scheme of the setup is shown in Supplementary Information Fig. S2. All the connection joints of the setup were carefully tested to be leak-free. The GO hollow fibre was sealed on a stainless-steel sample holder with epoxy resin, and mounted into the gas chamber of the setup. The chamber was swept by the testing gas for 3 minutes to remove impurity gases, and then pressurized by the gas to a target pressure. The inlet and outlet of the chamber were closed, leaving the GO hollow fibre membrane the only path for gas to leak/permeate. The pressure inside the gas chamber was monitored by the pressure transducer and was recorded by a computer. For gas permeation tests, the permeance of the gas through the membrane is calculated through the pressure-drop rate:


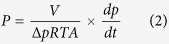


where *P* denotes the permeance (mol m^−2^ s^−1^ Pa^−1^); *V* is the volume of the gas chamber (m^3^); *Δp* is the pressure difference across the membrane (Pa), *R* is the gas constant (8.314 J mol^−1^ K^−1^); *T* is the measured temperature (K); *A* is the membrane surface area (m^2^); and *dp*/*dt* is the pressure drop rate.

The nanofiltration dye-rejection tests were conducted using a dead-end mode. The scheme of the testing setup is available in ref. 20. The GO hollow fibre membrane was mounted into a sample cylinder, in which a dye water solution was filled and pressurized to 5 bar. The permeate through the membrane was collected and analysed after the initial 24 hours to avoid possible interference from the adsorption on GO flakes. The dye concentration of the feed and the permeate were determined by a UV-spectrometer (UV-2101PC, Shimadzu, UK), and the rejection is calculated through the below definition:


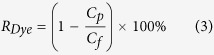


where *R*_*Dye*_ is the rejection of the GO membrane to the dye, 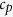 and 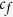 are the concentration of the permeate and the feed, respectively.

### Materials characterization

The morphology of the membranes was observed by a scanning electronic microscope (LEO Gemini 1525 FEGSEM). The surface morphology of the membranes was also observed with a Bruker Innova Atomic Force Microscope in tapping mode. XRD (XRD, X’pert Pro PANalytical) was used to determine the average interspace between GO flakes in GO membranes. Water contact angle of GO membrane were measured using a drope shap analyzer (DSA 10 MK2, Krüss GmbH, Hamburg, Germany). Flat GO membranes on PES substrate were used, instead of hollow fibres, in XRD, water contact angle measurement and AFM to ensure accurate diffraction angle and water contact angle measurements and a more uniform surface for AFM scanning. Ultraviolet–visible spectroscopy was used to determine the concentration of GO dispersions.

## Additional Information

**How to cite this article**: Chong, J. Y. *et al.* UV-Enhanced Sacrificial Layer Stabilised Graphene Oxide Hollow Fibre Membranes for Nanofiltration. *Sci. Rep.* **5**, 15799; doi: 10.1038/srep15799 (2015).

## Supplementary Material

Supplementary Information

## Figures and Tables

**Figure 1:**
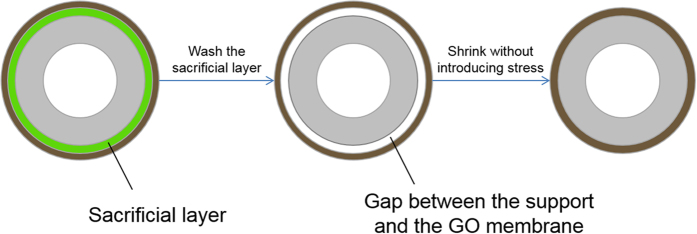
A scheme of the working principle of the use of sacrificial layer to stabilise GO membrane on a hollow fibre substrate.
