# Supplementary material for: UV-Enhanced Sacrificial Layer Stabilised Graphene Oxide Hollow Fibre Membranes for Nanofiltration
Source: Sci Rep. 2015 Nov 3;5:15799. doi: 10.1038/srep15799 (PMC4630626; doi:10.1038/srep15799)
Supplement: Supplementary Information [file srep15799-s1.pdf]

Supplementary Information

UV-Enhanced Sacrificial Layer Stabilised Graphene Oxide Hollow Fibre Membranes for Nanofiltration

J. Y. Chong,[1] N. F. D. Aba,[1] B. Wang,[1] C. Mattevi[2] and K. Li[1,*]

[1] Department of Chemical Engineering, Imperial College London, London SW7 2AZ, UK.

[2] Department of Materials, Imperial College London, London SW7 2AZ, UK.

* kang.li@imperial.ac.uk

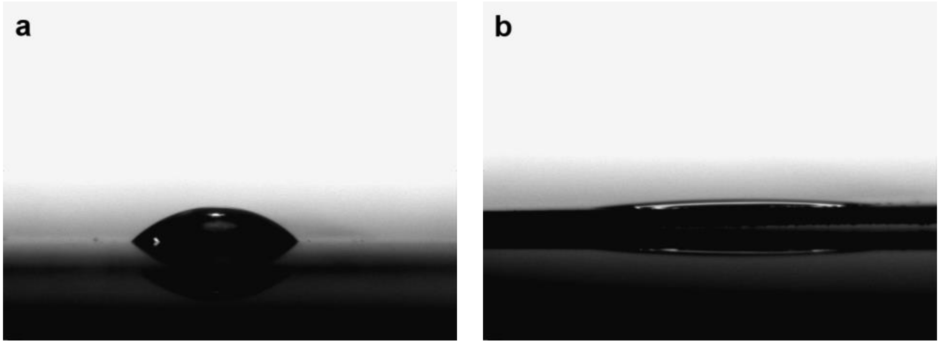

Figure S1: Water contact angles of GO membranes (a) before and (b) after UV treatment (2 hours). GO membranes became superhydrophilic after UV treatment.

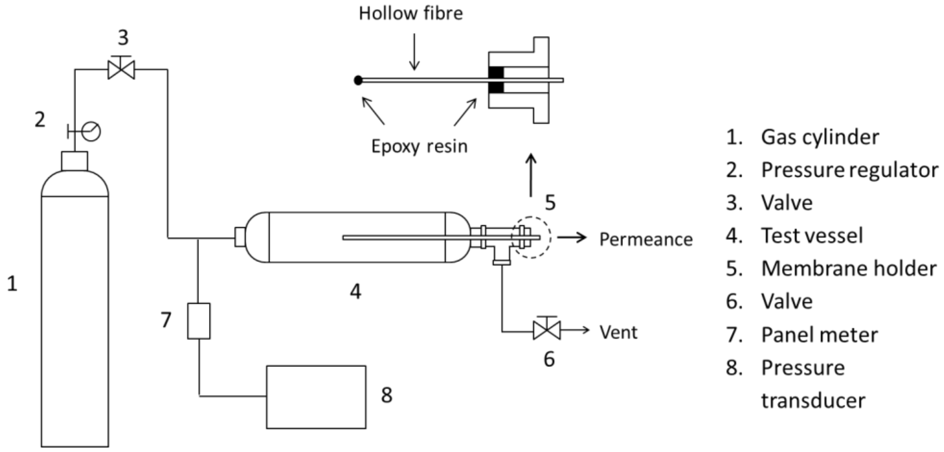

Figure S2. Scheme of the gas-tightness/gas-permeation setup used in this study.
